# Effects of High-Intensity Exercise Training on Adipose Tissue Mass, Glucose Uptake and Protein Content in Pre- and Post-menopausal Women

**DOI:** 10.3389/fspor.2020.00060

**Published:** 2020-06-17

**Authors:** Camilla M. Mandrup, Caroline B. Roland, Jon Egelund, Michael Nyberg, Lotte Hahn Enevoldsen, Andreas Kjaer, Andreas Clemmensen, Anders Nymark Christensen, Charlotte Suetta, Ruth Frikke-Schmidt, Betina Bernhard Utoft, Jonas Møller Kristensen, Jørgen F. P. Wojtaszewski, Ylva Hellsten, Bente Stallknecht

**Affiliations:** ^1^Department of Biomedical Sciences, University of Copenhagen, Copenhagen, Denmark; ^2^Department of Nutrition, Exercise and Sports, University of Copenhagen, Copenhagen, Denmark; ^3^Department of Clinical Physiology, Nuclear Medicine & PET, Rigshospitalet, Copenhagen, Denmark; ^4^Department of Applied Mathematics and Computer Science, Technical University of Denmark, Copenhagen, Denmark; ^5^Geriatric Research Unit, Herlev-Gentofte & Frederiksberg-Bispebjerg Hospitals, University of Copenhagen, Copenhagen, Denmark; ^6^Department of Clinical Medicine, University of Copenhagen, Copenhagen, Denmark; ^7^Department of Clinical Biochemistry, Rigshospitalet, Copenhagen, Denmark

**Keywords:** white adipose tissue metabolism, menopause, insulin sensitivity, lipid metabolism, glucose metabolism, mitochondrial enzymes

## Abstract

The menopausal transition is accompanied by changes in adipose tissue storage, leading to an android body composition associated with increased risk of type 2 diabetes and cardiovascular disease in post-menopausal women. Estrogens probably affect local adipose tissue depots differently. We investigated how menopausal status and exercise training influence adipose tissue mass, adipose tissue insulin sensitivity and adipose tissue proteins associated with lipogenesis/lipolysis and mitochondrial function. Healthy, normal-weight pre- (*n* = 21) and post-menopausal (*n* = 20) women participated in high-intensity exercise training three times per week for 12 weeks. Adipose tissue distribution was determined by dual-energy x-ray absorptiometry and magnetic resonance imaging. Adipose tissue glucose uptake was assessed by positron emission tomography/computed tomography (PET/CT) by the glucose analog [18F]fluorodeoxyglucose ([18F]FDG) during continuous insulin infusion (40 mU·m^−2^·min^−1^). Protein content associated with insulin signaling, lipogenesis/lipolysis, and mitochondrial function were determined by western blotting in abdominal and femoral white adipose tissue biopsies. The mean age difference between the pre- and the post-menopausal women was 4.5 years. Exercise training reduced subcutaneous (~4%) and visceral (~6%) adipose tissue masses similarly in pre- and post-menopausal women. Insulin-stimulated glucose uptake, assessed by [18F]FDG-uptake during PET/CT, was similar in pre- and post-menopausal women in abdominal, gluteal, and femoral adipose tissue depots, despite skeletal muscle insulin resistance in post- compared to pre-menopausal women in the same cohort. Insulin-stimulated glucose uptake in adipose tissue depots was not changed after 3 months of high-intensity exercise training, but insulin sensitivity was higher in visceral compared to subcutaneous adipose tissue depots (~139%). Post-menopausal women exhibited increased hexokinase and adipose triglyceride lipase content in subcutaneous abdominal adipose tissue. Physical activity in the early post-menopausal years reduces abdominal obesity, but insulin sensitivity of adipose tissue seems unaffected by both menopausal status and physical activity.

## Introduction

Adipose tissue redistributes during the menopausal transition, leading to an accumulation of abdominal subcutaneous and visceral adipose tissue. This might be a consequence of the hormonal shift with loss of estrogens during menopause (Davis et al., 2012), but how adipose tissue metabolism is affected by menopause is sparsely investigated. Systemic glucose homeostasis and skeletal muscle insulin sensitivity are positively affected by estrogens (Louet et al., 2004; Barros and Gustafsson, 2011). In support of this, we previously found a lower insulin-stimulated glucose uptake in skeletal muscle after menopause (Mandrup et al., 2018). Importantly, a similar increase in skeletal muscle glucose uptake in pre- and post-menopausal women was seen after high-intensity exercise training (Mandrup et al., 2018). We have also observed an increase in insulin-stimulated glucose uptake in skeletal muscle of overweight males after 3 months of exercise training, whereas adipose tissue glucose uptake remained unchanged or even decreased (Reichkendler et al., 2013). Whether exercise training affects insulin-stimulated glucose uptake in adipose tissue of pre- and post-menopausal women is not known.

Studies in rodents have shown that estrogens reduce the transcription of lipoprotein lipase and thereby lipid uptake and storage in adipose tissue (Homma et al., 2000; D'Eon et al., 2005). Those mechanisms also seem to apply to humans, as lipoprotein lipase activity increased after an oral glucose tolerance test in post- compared to pre-menopausal women (Santosa and Jensen, 2013). In addition, the post-menopausal women had higher storage of free fatty acids (FFA) in adipose tissue (Santosa and Jensen, 2013). Estrogens seem to decrease lipolysis through up-regulation of adipocyte α2-adrenergic receptors, thereby preventing an excess of FFA delivered to and deposited in ectopic tissues, such as liver and skeletal muscles (Kim et al., 2014). The effect of estrogens on adipose tissue lipolysis show regional differences, as estrogens blunt β-adrenergic stimulation of lipolysis in the abdominal but not in the gluteal region in pre-menopausal women (Gavin et al., 2013).

Studies of adipose tissue mitochondrial function are sparse, and changes relating to exercise training in combination with menopausal transition have not been investigated. We previously demonstrated higher mitochondrial activity in adipose tissue after 10 weeks of swim training in rodents (Stallknecht et al., 1991), but no studies of human mitochondrial function have shown positive adaptations to exercise training in adipose tissue (Camera et al., 2010; Larsen et al., 2015; Dohlmann et al., 2018).

The first aim of the present study was to investigate the impact of menopausal status on insulin-stimulated glucose uptake and proteins associated with glucose and lipid metabolism in abdominal and femoral white adipose tissue depots. Secondly, we aimed to study the effects of exercise training on white adipose tissue mass, glucose uptake and adipose tissue protein content in pre- and post-menopausal women. Moreover, we investigated differences in metabolism between the abdominal and femoral adipose tissue depots. We hypothesized that menopause would promote abdominal adipose tissue anabolism and that exercise training would reduce adipose tissue mass in both pre- and post-menopausal women.

## Methods

### Ethical Approval

Information about the study, including risks and discomforts associated with participation, was given in writing and orally before the participants gave their written consent to participate. The study was conducted according to the Helsinki Declaration, approved by the Ethical Committee in the capital region of Denmark, protocol no. H-1-2012-150 and preregistered at clinicaltrials.gov (no. NCT02076932).

### Participants and Study Design

Forty pre-menopausal and 39 post-menopausal women were included in the Copenhagen Women Study—Menopause. The study design has been described previously (Mandrup et al., 2017). In brief, the inclusion criteria were; healthy, sedentary, normal to overweight [Body Mass Index (BMI) 18.5–30.0 kg/m^2^], 45–57 years of age, either pre-menopausal [regular menses and plasma estradiol in the normal fertile range; follicular phase 0.05–0.51 nmol/l, mid cycle 0.32–1.83 nmol/l, luteal phase 0.16–0.78 nmol/l, and plasma follicle stimulating hormone (FSH) <20 IU/l] or post-menopausal (no menses for at least 1 year, estrogen <0.20 nmol/l and FSH 22–138 IU/l). Sedentary behavior was defined as not engaging in planned physical activities and performing less than a total of 2 h of physical activity per week during the last 2 years. A sedentary lifestyle was supported by maximal oxygen uptake <40 ml O_2_/min/kg at inclusion. Exclusion criteria were; smoking, use of hormonal contraception or replacement treatment, excessive alcohol intake, chronic disease, daily intake of medication or abnormal blood samples (screening for liver, kidney, and bone marrow function). Fasting abdominal and femoral adipose tissue biopsies were obtained from all participants at baseline and after the training intervention. A subgroup of 21 pre-menopausal and 20 post-menopausal women underwent a hyperinsulinemic euglycemic clamp, positron emission tomography/computed tomography (PET/CT) scan, and magnetic resonance imaging (MRI) scan. A biopsy from the abdominal and femoral adipose tissue was taken during insulin stimulation from participants of this subgroup. Skeletal muscle insulin sensitivity of this subgroup has been published (Mandrup et al., 2018). All participants were tested before and after 3 months of high-intensity aerobic exercise training, performed as supervised indoor bike exercise training three times a week. The duration of the training sessions was ~53 min and consisted of warm-up, three blocks of different intensities with multiple periods of maximal performance, and a cool down period. Training intensity was monitored by heart rate monitors (FT2, Polar, Finland).

### Experimental Methods

*Maximal oxygen* uptake was assessed by an incremental bicycle ergometer (Monark, Ergomedic 839 E, Sports & Medical, GIH, Sweden) protocol to exhaustion as previously described (Mandrup et al., 2017).

*Body composition* was assessed by dual-energy X-ray absorptiometry (DXA) scanning (Lunar iDXA, GE Healthcare, US) after an overnight fast. Additionally, abdominal and femoral adipose tissue depots were assessed by MRI on a 1.5 T Avanto (SIEMENS, Erlangen, Germany). Abdominal and femoral axial T1-weighted images were obtained using breath-hold (water suppression, slice thickness 6 mm, spacing between slides 7.2 mm, pixel size 1.1719 × 1.1719 mm). All scans were performed by the same investigator, who was blinded for menopausal status.

*Abdominal and femoral adipose tissue mass* was assessed by automated segmentation of the MRI using in-house developed software written in MATLAB (MathWorks, Natick, Massachusetts, United States). The visceral adipose tissue was delineated by the slice containing the disc between Th11 and Th12 (upper) and the most distal slice without the iliac crest (lower). The liver was manually segmented in ITK-SNAP (Yushkevich et al., 2006) and removed from the intra-abdominal segmentation. The abdominal subcutaneous adipose tissue limitation was the disc between Th12 and L1 (upper) and the last slide not including caput femoris (lower). In the femoral scan, the adipose tissue depots were delimited by the lower delimitation of the gluteal adipose tissue (upper) and the last slide without patella (lower). All images were bias-corrected (Larsen et al., 2014) and the adipose tissue depots were automatically delineated by unrolling the images and using the graph-cut method (Li et al., 2006). The inter-muscular/visceral adipose tissue depots were delineated by thresholding, with a threshold value determined by taking the median value of a 5-component k-means clustering on the muscular/abdominal compartment. Due to the T1 weighted sequence, femur was not visible at the femoral images, but the bone marrow was delineated separately from the inter-muscular fat. The adipose tissue volumes were converted to mass using a density of adipose tissue of 0.9 kg/L. Two abdominal and three femoral scans were excluded due to errors in the acquisition or movement artifacts.

*A hyperinsulinemic euglycemic clamp was* performed as previously described (DeFronzo et al., 1979; Mandrup et al., 2018). In brief, the pre-menopausal women were investigated in the late follicular phase (days 8–13) where plasma estrogens are high, but plasma progesterone low. All participants arrived at the laboratory after an overnight fast, without having engaged in physical activity or consumed caffeine for 36 h. Arterialized venous blood samples were obtained from a hand vein. Insulin were administered through an antecubital vein at a rate of 40 mU·m^−2^·min^−1^ together with variable rates of glucose to maintain euglycemia. Blood samples were obtained at baseline and every 30 min in pre-coated tubes (BD Vacutainer®, Becton, Dickinson and Company, Franklin Lakes, New Jersey, USA), immediately stored on ice and centrifuged at 4,000 rpm in 10 min before the plasma was stored at −80°C for later analyses of FFA and glycerol (clinical chemistry analyzer, Pentra C 400, Horiba, Kyoto, Japan) by investigators blinded for menopausal status. Estradiol and testosterone (competitive electrochemiluminescence immunoassay (ECLIA), Cobas 8000, e602 module, F. Hoffmann-La Roche Ltd., Rotkreuz, Switzerland) and sex hormone binding globulin (SHBG) (sandwich chemiluminescent immunometric method, Siemens Immulite 2000 XPi) were measured before insulin infusion. The participants were maintained in clamped (hyperinsulinemia, euglycemia) condition during the PET/CT scan.

### PET/CT Scan: Tracer, Image Acquisition, and Processing

PET/CT scans were performed on a Biograph mCT 128-slice (Siemens, Erlangen, Germany) as described previously (Mandrup et al., 2018). Briefly, a low-dose CT scan (120 kV, 11 mA) was performed for attenuation correction and anatomic localization prior to the PET scan. A low dose of [^18^F]fluorodeoxyglucose ([^18^F]FDG) (200 MBq) was injected intravenously precisely at the start of acquisition. The PET/CT scan included 60 min of dynamic acquisition of the abdomen followed by 2 × 10 min acquisition of the femoral region. The abdominal data was histogrammed into 26 timeframes of varying length (12·10, 4·120, 10·300 s) and reconstructed using the ordered subset expectation maximum algorithm with a point spread function correcting method (TrueX, Siemens), 336·336 pixels, with 8 iterations and 21 subsets. The femoral images were reconstructed as a single time frame (size: 400·400·174, voxel spacing: 2·2·2 mm, 3 iterations, and 21 subsets). All PET images were corrected for attenuation and scatter, and decay corrected to scan start. All PET/CT image analysis were performed using PMOD 3.304 (PMOD Technologies, Zurich, Switzerland) by an investigator blinded to menopausal and exercise training status. The [^18^F]FDG blood concentration was determined by image derived input function (IDIF) (Christensen et al., 2014) from a cylindrical volume of interest (VOI) in aorta at the level of L2/L3 The subcutaneous abdominal (anterior and posterior) and gluteal adipose tissue depots were defined at the CT scan with a segmentation using connected threshold with Hounsfield units from −150 to −50. The visceral adipose tissue was defined in the same way, inside a spherical VOI manually inserted in the visceral adipose tissue area. A two-pixel erosion was applied to all VOIs to avoid spillover from the intestinal walls. All VOIs were transferred to the PET images to obtain the metabolic rate of glucose (MR_glucose_), which was calculated as:

$$\begin{array}{l} MR_{glucose}=\frac{1,000\;\cdot K_{i}\cdot {glucose\;concentration\;}_{plasma}}{Lumped\;Constant\;\cdot density_{tissue}} \end{array}$$

The lumped constant (LC) accounts for differences in transport and phosphorylation rates between D-glucose and 2-fluoro-2-deoxy-D-glucose and transforms the [^18^F]FDG uptake rate to glucose uptake rate. The lumped constant of adipose tissue was set to 1.14 (Virtanen et al., 2001) and the density of adipose tissue was set to 0.9 g/ml. The K_*i*_ is the [^18^F]FDG influx rate constant (ml blood·ml tissue^−1^·min^−1^) and was derived from a mathematic model fit to the dynamic time activity curve in PMOD. To estimate K_*i*_ from the static scan, the mean activity of the defined VOI is divided by the [^18^F]FDG blood concentration. This value is extrapolated from the area under the curve of the IDIF from 0 to 60 min to the middle of the static scan (60 min + 10 min) in MATLAB.

$$\begin{array}{l} {estimated\;K}_{i\;}=\;\frac{\;{Activity\;}_{mean\;activity\;of\;the\;VOI\;\left[{kBq\cdot ml_{tissue}}^{-1}\right]}}{AUC_{available\;FDG\;i\;the\;blood\;\left[\;\frac{kBq\cdot \min}{ml_{blood}}\;\right]}} \end{array}$$

*Abdominal and femoral adipose tissue biopsies* were obtained both in the overnight fasted state and during insulin stimulation 120 min after initiation of the clamp on two separate test days. After local anesthesia (Lidocaine 20 mg/ml, SAD, Copenhagen, Denmark), the biopsies were obtained *ad modum* Bergström (Bergstrom, 1975) and immediately frozen in liquid nitrogen and stored at −80°C for later analyses.

*Adipose tissue preparation and western blotting* was performed to investigate protein content as previously described (Kristensen et al., 2007). Briefly, adipose tissue homogenates were rotated at 4°C for 60 min, centrifuged 40 min at 16,000 g, and the infranatant was harvested as the adipose tissue lysate. Total protein content in adipose tissue lysates was determined by the bicinchoninic acid method (PierceBiotechnology, Inc. Rockford, IL, USA). Lysates were solubilized in Laemmli sample buffer, and an equal amount of total protein from each sample was separated by SDS-page and transferred to PVDF-membranes. Membranes were blocked for 10 min in 2–3% skimmed milk or 3% BSA in TBS containing 0.05% Tween-20 (TBST buffer) and incubated overnight at 4°C in primary antibodies against glucose transporter 4 (GLUT4) [Thermo Fisher Scientific (# PA1-1065), MA, USA, diluted 1:1,000], akt substrate 160 kDa (AS160) [Millipore (#07-741), MA, USA, diluted 1:1,000], hexokinase (HK) [Cell Signaling Technology (#2106), MA, USA, diluted 1:1,000], cluster of differentiation 36 (CD36) [R&D systems (#AF2519), MN, USA, diluted 1:1,000], acetyl-CoA carboxylase (ACC) [Streptavidin-HRP, DAKO (#P0397), Denmark, diluted 1:2,000], adipose triglyceride lipase (ATGL) [Cell Signaling (#2138), MA, USA, diluted 1:1,000], citrate synthase (CS) [Abcam (#ab96600), Cambridge, UK, diluted 1:3,000) and oxidative phosphorylation (OXPHOS) complex 1–5 [Abcam (#ab110411), Cambridge, UK, diluted 1:10,000]. Membranes were incubated with horseradish peroxidase (HRP) conjugated secondary antibodies (Jackson ImmunoResearch, West Grove, PA, USA, diluted 1:5,000) for 45 min at room temperature before visualizing protein bands with Luminata Forte Western HRP Substrate [Millipore (#WBLUF0500), MA, USA] and ChemiDoc MP imaging system (BioRad, CA, USA). Protein signals were quantified (Image Lab version 4.0) and protein content levels expressed as arbitrary units. Protein content used for statistical analysis are expressed as a ratio to total protein content in the sample as measured by stain-free protein technology.

*Statistical analysis* was performed using SAS Enterprise Guide 7.1 (©SAS Institute Inc., Cary, NC, USA) and graphic presentations was made using GraphPad Prism 7.00 (GraphPad Software, Inc., La Jolla, CA, USA). Prior to inclusion, power calculations regarding insulin-stimulated glucose uptake measured by [^18^F]FDG PET/CT and adipose tissue masses measured by MRI revealed that inclusion of 20 pre- and 20 post-menopausal women would enable detection of a 21 and a 20% change, respectively, with a likelihood of 80%. Descriptive statistics for parametric data are calculated by a two-way ANOVA and given as mean [95% confidence interval (CI)]. Masses of abdominal and femoral adipose tissue depots were analyzed by a 2-way ANCOVA with the number of analyzed slices as covariate. Adipose tissue glucose uptake determined by PET/CT and protein content determined by western blotting were assessed using a mixed model with group and time as variables and participant ID as repeated measures. Interactions between time and group were further analyzed for within group effects by ls-means. Protein content during fasting (all participants) are presented in Figures 4, 5, 7. The difference in protein content after 2 h of hyper insulin stimulation (subgroup of participants) are given as delta-values and only significant results are shown (Figures 6, 8). Statistical significance was set as *p* ≤ 0.05.

## Results

Eighteen late pre- and 20 early post-menopausal women with a difference of 4.5 years of age were included in the subgroup of participants who underwent the hyperinsulinemic euglycemic clamp, PET/CT and insulin stimulated adipose tissue biopsies (Table 1). One post-menopausal woman was excluded from post-testing due to an emerging neuro-muscular disease. Characteristics of all of the participants has previously been published (Mandrup et al., 2017). Time since last menstruation for the post-menopausal women was 3.2 (2.6–3.8) years at inclusion. Plasma estradiol/SHBG index and reflected the menopausal status and the index did not change after the intervention. FSH reflected menopausal status. The post-menopausal women had lower bioavailable testosterone levels than the pre-menopausal women (*p* = 0.04), and bioavailable testosterone tended (*p* = 0.06) to decrease similarly in both groups after the intervention. Cardiorespiratory fitness increased (*p* < 0.0001) similarly in both groups after the intervention (Table 1).

**Table 1:** Participant characteristics of pre- and post-menopausal women before and after 3 months of high-intensity training.

	**Pre-menopausal**		**Post-menopausal**	
	**Baseline**	**3 months**	**Baseline**	**3 months**
	**(*n* = 18)**	**(*n* = 18)**	**(*n* = 20)**	**(*n* = 19)**
Age	48.5 (47.5–49.4)		53.0 (51.6–54.4)	
Body weight (kg)	66.1 (62.4–69.8)	66.1 (62.4–69.4)	67.0 (62.8–71.2)	66.5 (61.9–71.2)
BMI	23.5 (22.4–24.6)	23.4 (22.4–24.5)	23.6 (22.5–24.8)	23.5 (22.3–24.8)
Fat mass (kg)^a^	22.6 (20.6–24.5)	22.1 (20.1–24.1)	23.6 (20.8–26.4)	22.5 (19.3–25.7)
Fat (%)^a^	34.1 (32.1–36.0)	33.4 (31.5–35.2)	34.9 (32.3–37.4)	33.3 (30.4–36.2)
Android fat (%)^a^	35.8 (31.7–39.8)	35.2 (31.0–39.4)	35.9 (30.7–41.1)	34.2 (28.4–40.0)
Gynoid fat (%)^a^	40.0 (38.0–41.9)	38.8 (37.1–40.6)	41.0 (38.9–43.1)	38.4 (36.0–40.8)
Android/gynoid ratio	0.89 (0.80–0.99)	0.91 (0.80–1.01)	0.86 (0.76–0.96)	0.87 (0.76–0.99)
Maximal oxygen uptake (ml O_2_/min)^a^	2085 (1952–2218)	2308 (2174–2442)	2018 (1887–2148)	2241 (2108–2374)
FSH (IU/L)^b^	12.6 (7.2–18.1)	12.8 (7.7–18.0)	96.0 (82.8–109.3)	90.7 (79.7–101.7)
Estradiol/SHBG index^b^	6.5 (4.3–9.8)	7.0 (4.6–10.6)	1.4 (1.1–1.7)	1.3 (1.1–1.6)
Bioavailable testosterone (ng/dL)^b^	5.3 (4.3–6.5)	4.9 (4.0–6.1)	4.0 (3.0–4.9)	3.7 (3.0–4.6)

*Data are presented as mean (95% confidence limits). Group differences and effect of the intervention were assessed by a two-way ANOVA*.

a *Change from baseline to 3 months: (p ≤ 0.05)*.

b *Difference between groups (p ≤ 0.05). BMI, Body Mass Index; FSH, Follicle stimulating hormone; SHBG, sex hormone binding globulin*.

### Adipose Tissue Mass

DXA scans: Whole body fat mass, fat percentage, and android and gynoid fat percentages did not differ between the pre- and the post-menopausal women (all: *p* > 0.05) and the masses were reduced similarly in the two groups with 3 months of high-intensity exercise training (*p* < 0.01, *p* < 0.001, *p* < 0.01, *p* < 0.0001, respectively) (Table 1). The android/gynoid ratio was similar in the two groups and did not change after the intervention.

**Figure 1:**
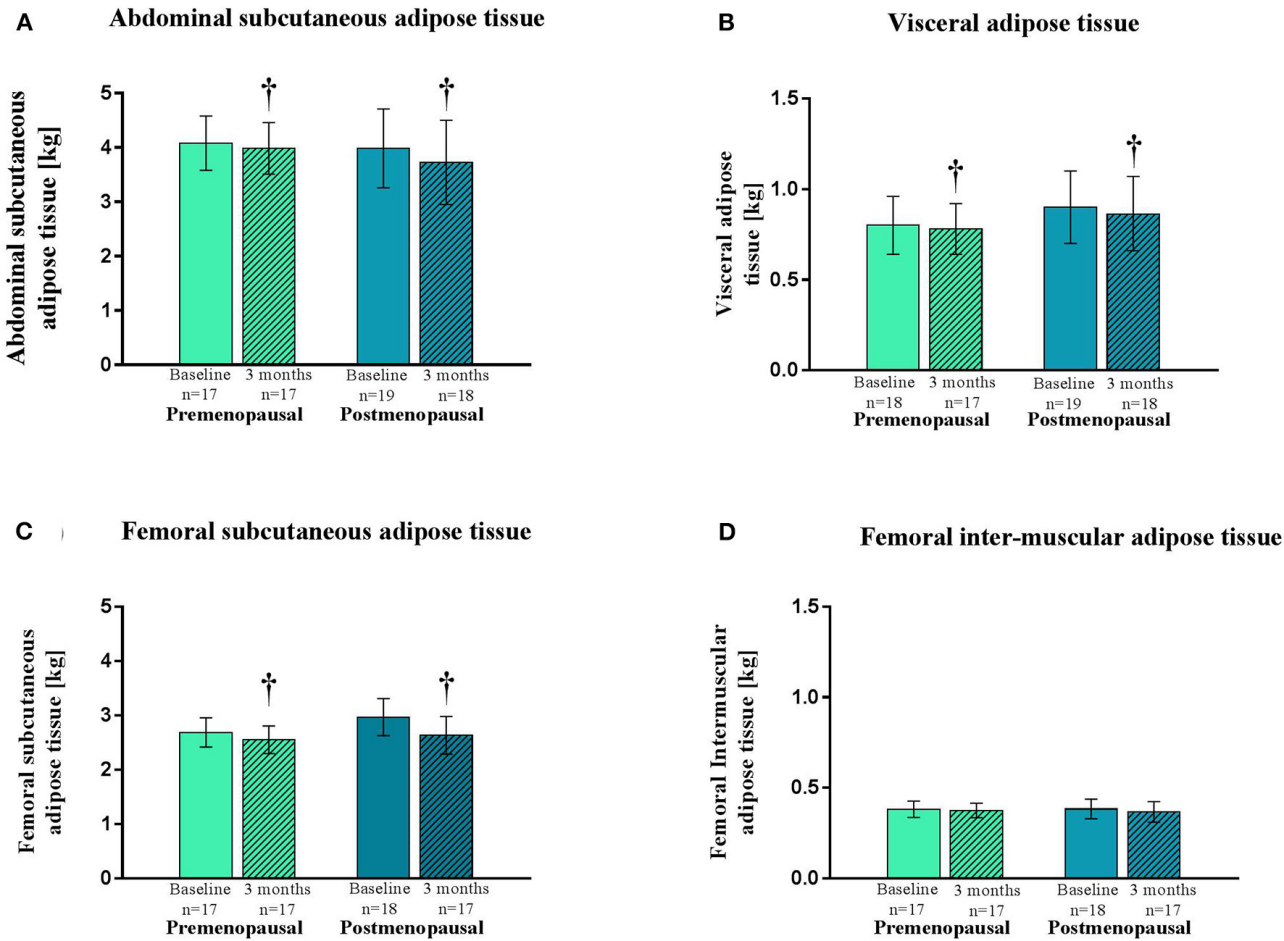
Abdominal and femoral adipose tissue depots as determined by magnetic resonance imaging in pre- and post-menopausal women before and after 3 months of high-intensity training. Data are presented as mean (95% confidence limits). Changes from baseline to 3 months †*p* ≤ 0.05. **(A)** Abdominal subcutaneous adipose tissue. **(B)** Visceral adipose tissue. **(C)** Femoral subcutaneous adipose tissue. **(D)** Femoral inter-muscular adipose tissue.

MRI scans: Abdominal subcutaneous and visceral adipose tissue masses did not differ between the pre- and the post-menopausal women and a dipose tissue masses decreased similarly in the two groups after the intervention (*p* < 0.005 and *p* = 0.03, respectively) (Figure 1). Also, femoral subcutaneous and inter-muscular adipose tissue mass did not differ between groups (Figure 1). The femoral subcutaneous adipose tissue was reduced similarly in the two groups after the intervention (*p* = 0.01), whereas the inter-muscular adipose tissue mass remained unchanged (Figure 1).

#### Insulin-Stimulated Glucose Uptake in Adipose Tissue

**Figure 2:**
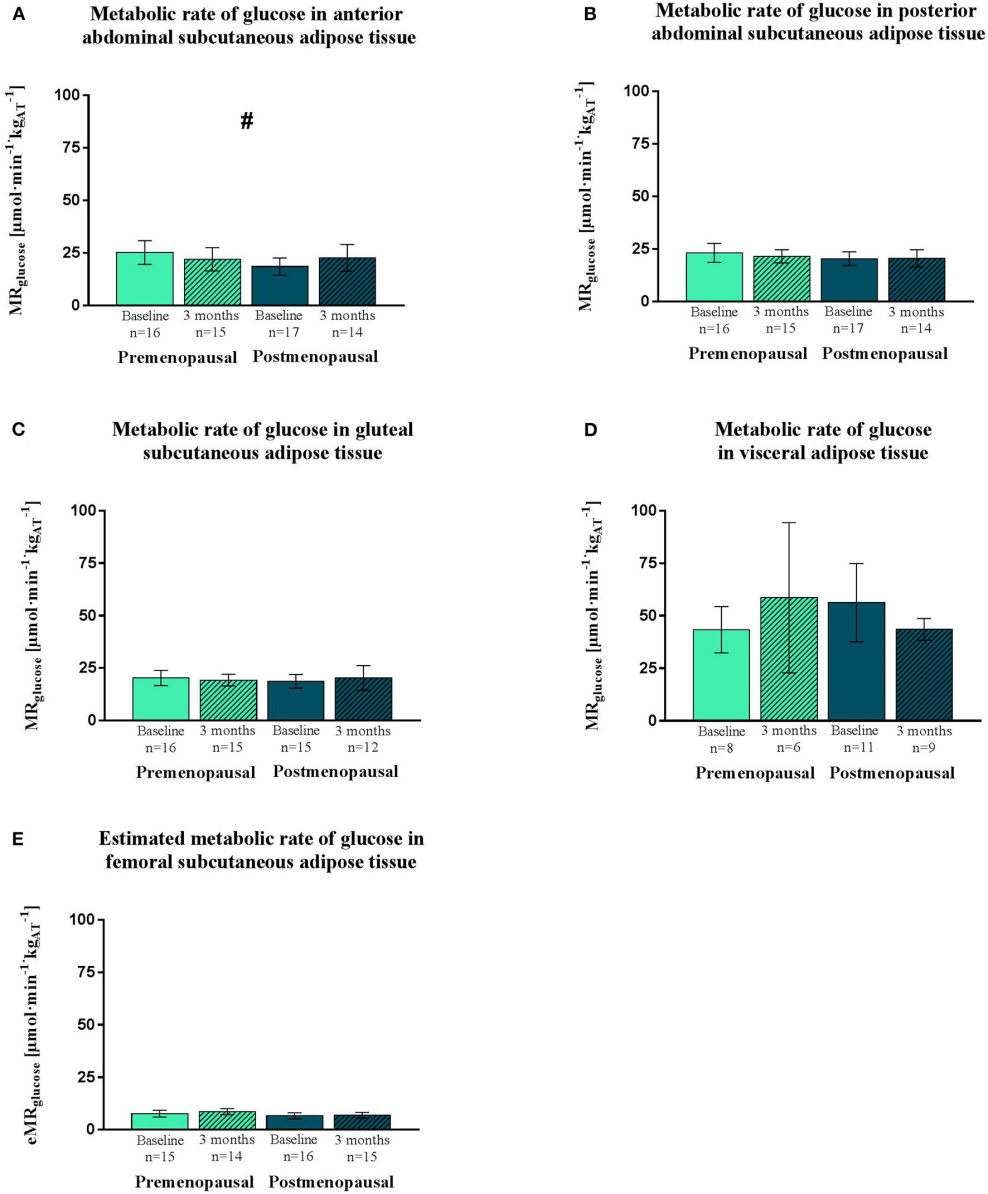
Insulin-stimulated glucose uptake in adipose tissue, expressed as MR_glucose_. PET/CT-derived data of MR_glucose_ of abdominal subcutaneous (anterior and posterior), visceral and gluteal adipose tissue. Estimated MR_glucose_ of femoral subcutaneous adipose tissue. Data was obtained during a hyperinsulinemic euglycemic clamp in pre- and post-menopausal women before and after 3 months of high-intensity training. Data are presented as mean (95% confidence limits). MR_glucose_ is higher in visceral than subcutaneous adipose tissue depots. Interaction between groups and time ^#^*p* ≤ 0.05. MR_glucose_, Metabolic rate of glucose. **(A)** Metabolic rate of glucose in anterior abdominal subcutaneous adipose tissue. **(B)** Metabolic rate of glucose in posterior abdominal subcutaneous adipose tissue. **(C)** Metabolic rate of glucose in gluteal subcutaneous adipose tissue. **(D)** Metabolic rate of glucose in visceral adipose tissue. **(E)** Estimated metabolic rate of glucose in femoral subcutaneous adipose tissue.

The insulin-stimulated glucose uptake was higher in visceral compared to all subcutaneous adipose tissue depots (all: *p* < 0.0001) (Figure 2). The training response differed (*p* = 0.02) between the pre- and the post-menopausal women as the glucose uptake in the anterior abdominal subcutaneous adipose tissue decreased in the pre- and increased in the post-menopausal women. *Post-hoc* analysis showed no within-group difference (Figure 2). No significant differences in insulin-stimulated glucose uptake between or within groups were observed in the posterior abdominal subcutaneous, visceral, gluteal, or femoral subcutaneous adipose tissue depots (Figure 2).

#### Plasma Free Fatty Acids and Glycerol

**Figure 3:**
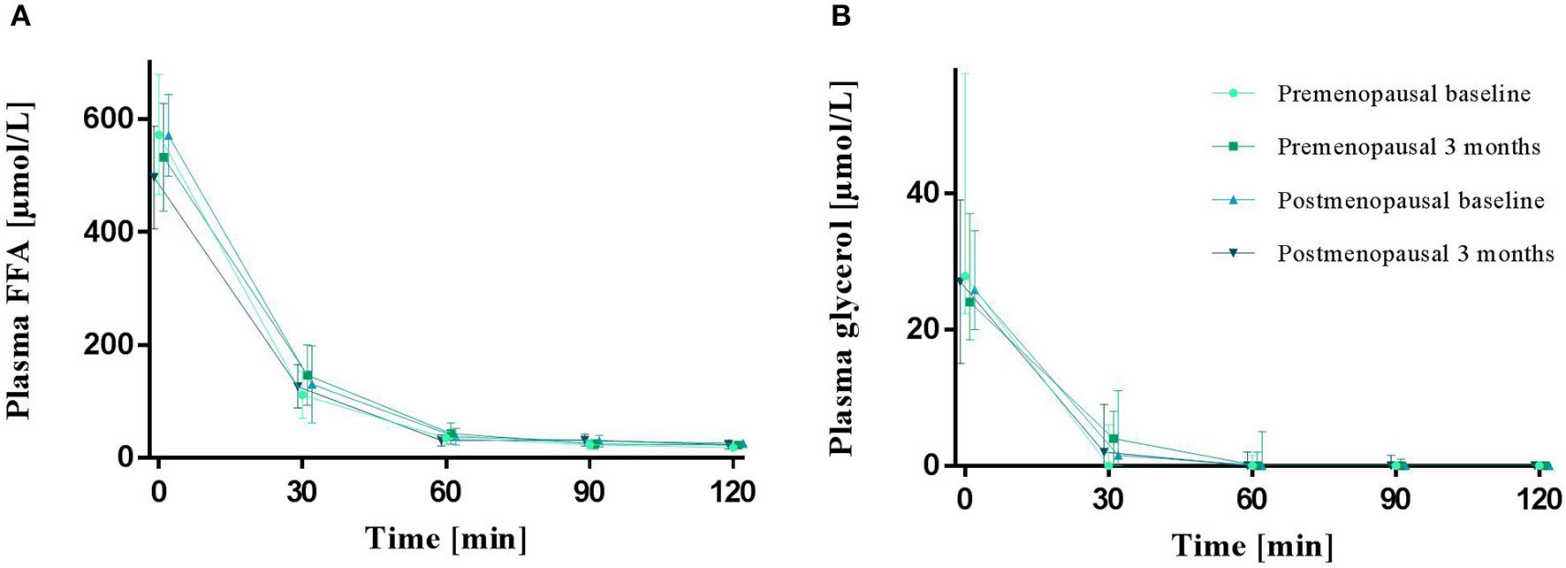
Plasma FFA **(A)** and glycerol **(B)** in the fasted state (0 min) and during a hyperinsulinemic euglycemic clamp in pre- and post-menopausal women before (baseline) and after 3 months of high-intensity training. Plasma FFA are presented as mean (95% confidence limits) and glycerol as median (25–75 percentile). FFA, Free fatty acids.

Plasma FFA and glycerol did not differ between pre- and post-menopausal women during fasting (0 min). In addition, the decline in plasma FFA and glycerol during hyperinsulinemic stimulation (30–120 min) was similar between groups. The exercise training intervention did not change fasting or insulin suppression of plasma FFA or glycerol (Figure 3).

#### Proteins Associated With Insulin Signaling and Glucose Metabolism (Figure 4)

In abdominal adipose tissue, HK content was higher in post- compared to pre-menopausal women (*p* = 0.03), and exercise training did not affect HK content. In femoral adipose tissue, exercise training led to no change in the pre-menopausal group but increased AS160 content in the post-menopausal group (interaction: *p* = 0.04, *post-hoc* analysis: within-group effect in post-menopausal women *p* = 0.02). Neither menopausal state nor exercise training were associated with the abundance of adipose tissue GLUT4.

**Figure 4:**
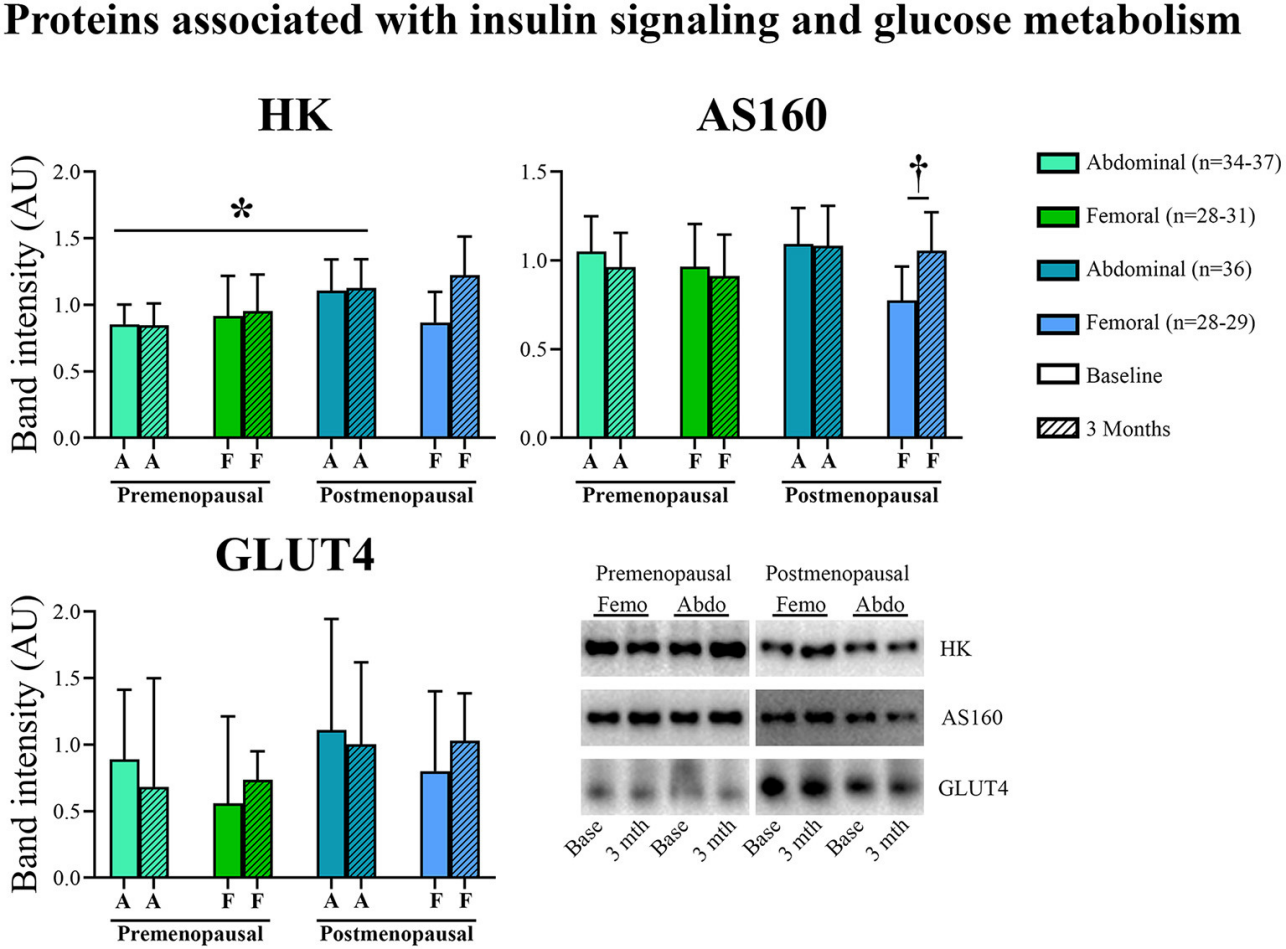
Abundance of proteins associated with insulin signaling and glucose metabolism in abdominal and femoral subcutaneous adipose tissue in pre- and post-menopausal woman before and after 3 months of high-intensity training. In abdominal adipose tissue, HK content was higher in post- compared to pre-menopausal women. In femoral adipose tissue, exercise training increased AS160 content in post-menopausal women. Changes from baseline to 3 months †*p* ≤ 0.05. Difference between groups *p ≤ 0.05. HK and AS160 protein abundances are presented as mean (95% confidence limits). GLUT4 protein abundance is presented as median (25–75 percentile). HK, Hexokinase; AS160, Akt substrate 160 kDa; GLUT4, Glucose transporter 4.

#### Proteins Associated With Lipogenesis and Lipolysis (Figure 5)

In abdominal adipose tissue, ATGL content was higher in post- compared to pre-menopausal women (*p* = 0.03). Further, the ATGL content was higher in abdominal compared to femoral adipose tissue in the post-menopausal women (*p* = 0.005). In femoral adipose tissue, insulin stimulation suppressed ATGL content in pre- but not in post-menopausal women (*p* = 0.05, Figure 6). In abdominal adipose tissue, exercise training resulted in a different response in pre- vs. post-menopausal women, as CD36 content decreased in pre- but increased in post-menopausal women (interaction: *p* = 0.02). Post hoc analyses detected no within-group effect. In contrast, exercise training increased the ACC content in abdominal adipose tissue in the pre-menopausal women and decreased ACC content in the post-menopausal women (interaction: *p* = 0.05). *Post-hoc* analyses showed a within-group increase (*p* = 0.05) in pre-menopausal women after the training intervention. In both groups, ACC content was higher in abdominal compared to femoral adipose tissue (pre-menopausal: *p* = 0.005, post-menopausal: *p* = 0.03).

**Figure 5:**
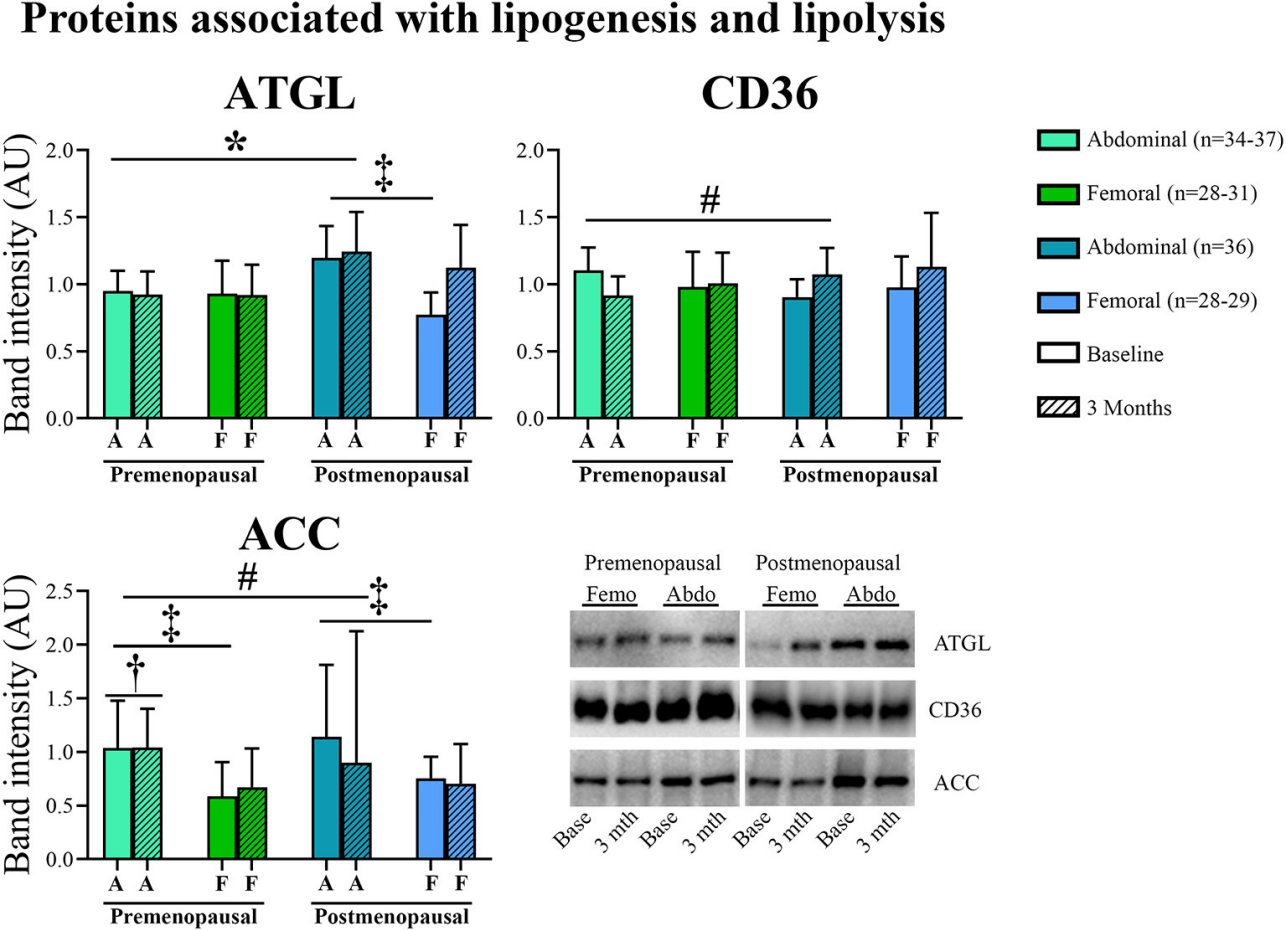
Abundance of proteins associated with lipogenesis and lipolysis in abdominal and femoral subcutaneous adipose tissue in pre- and post-menopausal woman before and after 3 months of high-intensity training. Abdominal ATGL content was higher in post- compared to pre-menopausal women. ATGL content was higher in abdominal compared to femoral adipose tissue in the post-menopausal women. Abdominal CD36 content decreased after exercise training in pre- but increased in post-menopausal women. Abdominal ACC content increased after exercise training in pre- and decreased in post-menopausal women. Pre-menopausal women increased abdominal ACC content after the training intervention. ACC content was higher in abdominal compared to femoral adipose tissue. Interaction between groups and time ^#^*p* ≤ 0.05. Changes from baseline to 3 months †*p* ≤ 0.05. Difference between abdominal and femoral depots^‡^*p* ≤ 0.05. Difference between groups *p ≤ 0.05. ATGL and CD36 protein abundances are presented as mean (95% confidence limits). ACC protein abundance is presented as median (25–75 percentile). ATGL, Adipose triglyceride lipase; CD36, Cluster of differentiation 36; ACC, Acetyl-CoA carboxylase.

**Figure 6:**
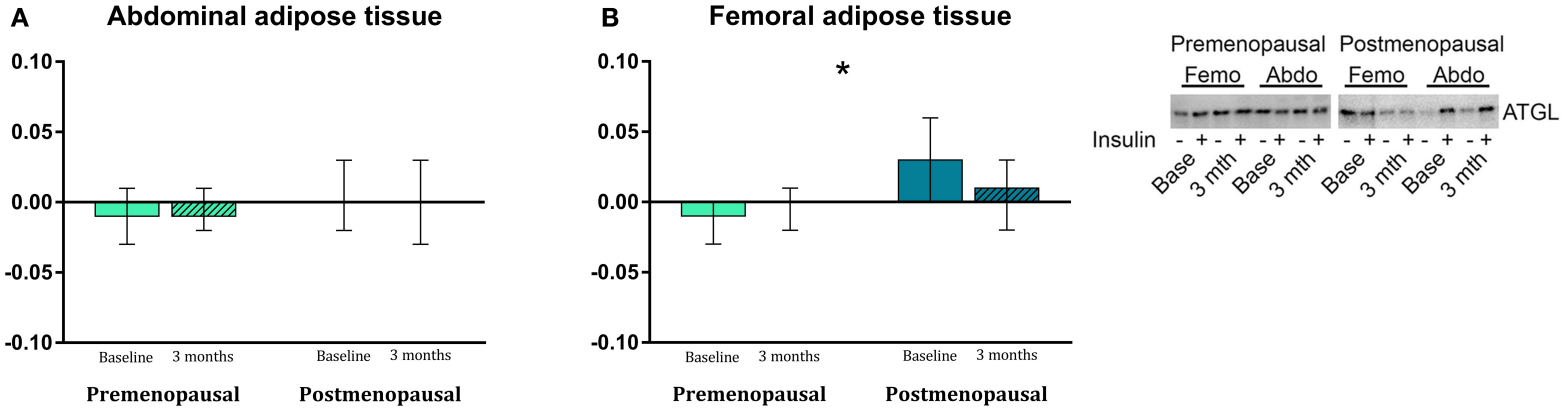
The effect of 120 min of hyper insulin stimulation on protein abundance of ATGL (delta value, arbitrary units) in abdominal **(A)** and femoral **(B)** adipose tissue in pre- and post-menopausal women before and after 3 months of high-intensity training. Insulin stimulation suppressed ATGL in femoral subcutaneous adipose tissue in pre- but not in post-menopausal women. Difference between groups *p ≤ 0.05. Data are presented as mean (95% confidence limits). ATGL, Adipose triglyceride lipase.

#### Proteins associated with mitochondria (Figure 7)

In post-menopausal women, the OXPHOS complex 1 content was higher in abdominal compared to femoral adipose tissue (*p* = 0.05). Insulin stimulation suppressed OXPHOS complex 3 content after exercise training in both pre- and post-menopausal women in abdominal (*P* = 0.04) and femoral (*p* = 0.05) adipose tissue (Figure 8). The effect of exercise training on OXPHOS complex 5 content in abdominal adipose tissue differed between the groups as it increased in post- but not in pre-menopausal women (interaction *p* = 0.04. *Post-hoc* analysis, within-group effect in post-menopausal women *p* = 0.03).

**Figure 7:**
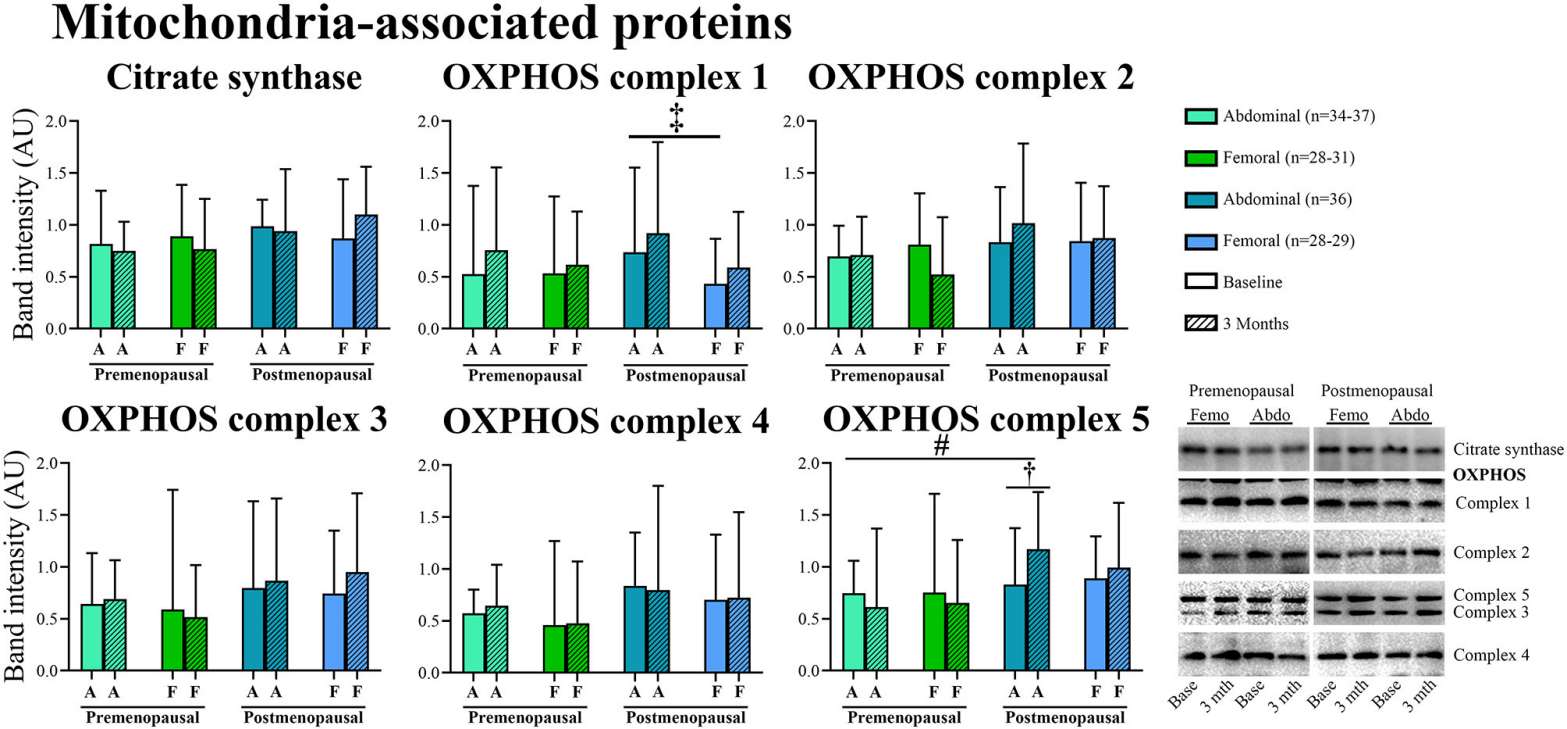
Abundance of proteins associated with mitochondria in abdominal and femoral subcutaneous adipose tissue in pre- and post-menopausal woman before and after 3 months of high-intensity training. Abundance of OXPHOS complex 1 was higher in abdominal compared to femoral adipose tissue in post-menopausal women. Abdominal OXPHOS complex 5 content increased after the training intervention in post- but not in pre-menopausal women. Difference between abdominal and femoral depots^‡^*p* ≤ 0.05. Interaction between groups and time ^#^*p* ≤ 0.05. Changes from baseline to 3 months †*p* ≤ 0.05. Data are presented as median (25–75 percentile). CS, Citrate synthase.

**Figure 8:**
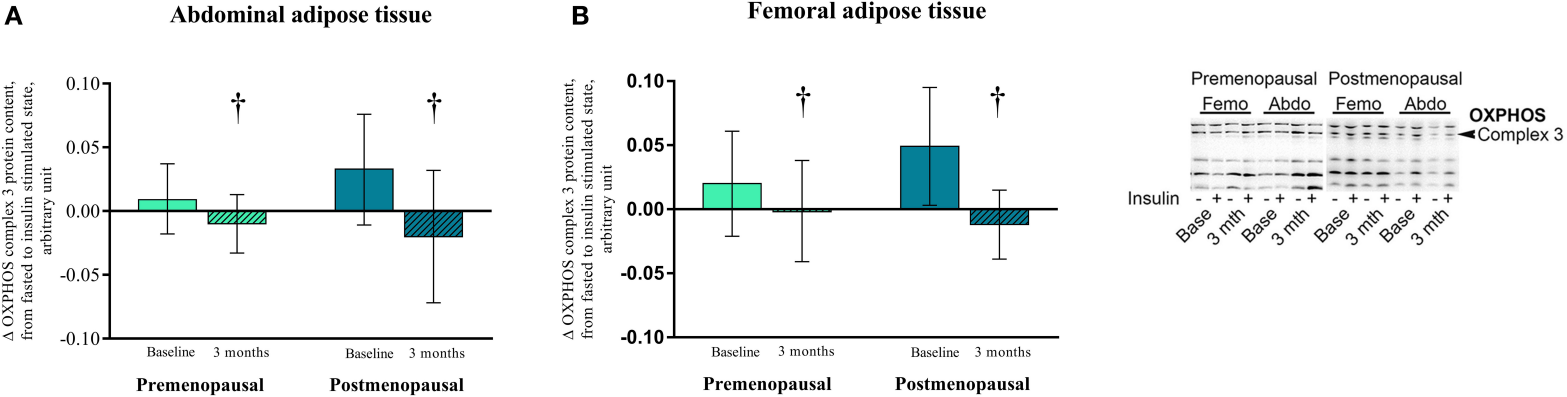
The effect of 120 min of hyper insulin stimulation on OXPHOS complex 3 (delta value, arbitrary units) in abdominal and femoral adipose tissue in pre- and post-menopausal women before and after 3 months of high-intensity training. Difference from baseline to 3 months †*p* ≤ 0.05. Data are presented as mean (95% confidence limits).

## Discussion

This study demonstrates that insulin-stimulated glucose uptake in abdominal, gluteal, and femoral adipose tissue depots are similar in pre- and post-menopausal women, despite skeletal muscle insulin resistance in post- compared to pre-menopausal women in the same cohort (Mandrup et al., 2018). Also, insulin-stimulated glucose uptake in adipose tissue depots was not altered markedly by 3 months of high-intensity exercise training in either group, whereas skeletal muscle insulin sensitivity increased after exercise training in both groups (Mandrup et al., 2018). Insulin sensitivity was higher in visceral compared to subcutaneous adipose tissue depots and the mass of visceral and subcutaneous adipose tissue depots diminished during the intervention to the same extent in pre- and post-menopausal women.

We are the first to investigate insulin-stimulated glucose uptake in adipose tissue of pre- and post-menopausal women by [^18^F]FDG PET/CT and our observation of similar glucose uptake in adipose tissue depots before and after menopause contributes to the understanding of physiological changes with the menopausal transition. Skeletal muscle is responsible for ~85% of whole-body insulin-stimulated glucose uptake (DeFronzo et al., 1979). In comparison, adipose tissue insulin-stimulated glucose uptake measured by PET/CT in non-obese and moderately obese men (BMI: 19–24 and 27–34 kg/m^2^, respectively) has been reported to comprise 2.6 and 4.2%, respectively, of the total glucose disposal (Virtanen et al., 2002). In morbidly obese individuals (BMI > 40 kg/m^2^), the insulin-stimulated glucose uptake in adipose tissue has been reported to contribute substantially to total glucose uptake, accounting for up to 20% of glucose disposal (Gniuli et al., 2010). Clearly, a larger adipose tissue mass yields a larger glucose uptake. In addition, hypothetically, insulin resistance in skeletal muscle could cause subsequent spillover of glucose to adipose tissue. Insulin resistant subjects had a diminished fractional uptake of glucose in skeletal muscle compared to insulin sensitive subjects, but the fractional uptake of glucose in abdominal, femoral, and intraperitoneal adipose tissue depots was comparable between insulin resistant and insulin sensitive subjects (Ferrannini et al., 2018). This is consistent with our findings, where skeletal muscle insulin sensitivity was diminished in post- compared to pre-menopausal women (Mandrup et al., 2018) with no influence of menopausal state on glucose uptake in visceral or subcutaneous abdominal or femoral adipose tissue.

We found no change in adipose tissue insulin sensitivity after exercise training in either pre- or post-menopausal women. In young overweight men, we previously found that insulin-stimulated glucose uptake in various adipose tissue depots, as determined by [^18^F]FDG PET/CT, was similar or even decreased after 3 months of exercise training (Reichkendler et al., 2013). In addition, 3 months of endurance training did not influence adipose tissue glucose uptake in healthy men with or without a family history of type 2 diabetes as determined by microdialysis or *in vitro* (Dela and Stallknecht, 2010). However, in a cross-sectional study we found higher insulin sensitivity in adipose tissue of endurance-trained compared to sedentary young men (Stallknecht et al., 2000) and another cross-sectional study showed increased insulin-stimulated glucose transport in adipocytes from trained compared to sedentary humans (Rodnick et al., 1987). Also, in rodents, it has been shown, that high-capacity running rats have a higher glucose uptake in brown and visceral adipose tissue, and tend to show a higher uptake in subcutaneous adipose tissue compared to low-capacity running rats (Park et al., 2015). However, ovariectomy in both groups of rats had no effect on glucose uptake in adipose tissue. Hence, insulin-stimulated glucose uptake in adipose tissue seems to be increased in trained individuals, whereas 3 months of exercise training or menopausal status do not influence adipose tissue insulin sensitivity.

Glucose uptake has previously been reported to be higher in visceral compared to subcutaneous adipose tissue, both during fasting (4–6 h) (Christen et al., 2010) and steady-state insulin infusion measured by [^18^F]FDG PET/CT in gender mixed cohorts (Ng et al., 2012) and in obese men (Reichkendler et al., 2013). This implies a higher metabolic activity in the visceral adipose tissue, which also applies to the pre- and post-menopausal women of the present study. Visceral adiposity is associated with metabolic dysregulation, although the mechanisms are not clear (Després et al., 2008). Visceral adipose tissue mass increases with age, but the consequences of menopause *per se* on visceral adipose tissue accumulation is debated (Kuk et al., 2009). We observed no difference in visceral adipose tissue mass between non-obese pre- and post-menopausal women.

Exercise training is known to reduce abdominal obesity (Ross and Després, 2009), but the effect in post-menopausal women is not clear. A cross-sectional study reported that post-menopausal women had a higher visceral adipose tissue mass compared to pre- and perimenopausal women, although the three groups were comparable with respect to subcutaneous adipose tissue mass (Dugan et al., 2010). Within the subgroups of the pre-, peri-, and post-menopausal women, who were physically active, the post-menopausal women still had significantly increased visceral adipose tissue depots, proposing that post-menopausal women had a blunted response to exercise training in terms of reducing visceral adipose tissue mass. Although it has been reported that post-menopausal women are able to reduce visceral adipose tissue mass after a 1-year aerobic exercise intervention (Friedenreich et al., 2011), there was no dose-response effect in reducing visceral depots (Friedenreich et al., 2015). We did not observe a reduced capability of post- compared to pre-menopausal women to decrease the visceral adipose tissue mass after 3 months of high-intensity exercise training, suggesting that physical activity in the early post-menopausal years can contribute to reduced visceral adipose tissue mass.

Hexokinase is an important enzyme that phosphorylates glucose initially after cellular uptake, before storage as glycogen, or consumption via glycolysis or the pentose phosphate pathway. The protein content of HK was higher in the abdominal adipose tissue of the post- compared to the pre-menopausal women and, as expected, no increase was observed after 120 min of insulin stimulation. Previously, we demonstrated an increase in HK content in skeletal muscle in post- compared to pre-menopausal women of the same cohort (Mandrup et al., 2018), suggesting that protein content of HK is upregulated in both skeletal muscle and abdominal adipose tissue after menopause, perhaps to overcome insulin resistance. It has been shown that HK mRNA in adipose tissue is upregulated after 180 min of insulin stimulation in metabolically healthy and obese men and women, but not in type 2 diabetes patients, suggesting that acute insulin dependent upregulation of HK is impaired due to or maybe as a path in adipose tissue insulin resistance (Ducluzeau et al., 2001).

Traditionally, adipose tissue insulin sensitivity is evaluated as insulin suppression of adipose tissue lipolysis, measured by reduced release of FFA to the blood. This measure is relevant as FFA are the most metabolically important products of adipose tissue lipolysis and high plasma concentrations are linked to muscle and liver insulin resistance, hypertriglyceridemia, and impaired vascular function (Jensen, 2007). We demonstrated no difference in FFA suppression between pre- and post-menopausal women, and no change after high-intensity exercise. This is opposite to a study of mix-gender, upper body obese (BMI 28–36 kg/m^2^) but non-diabetic subjects, where 40 weeks of combined diet and exercise intervention let to ~50% lower plasma oleate concentration during a steady-state hyperinsulinemic clamp (Shadid and Jensen, 2006). Our participants were metabolically healthy which might explain the absence of differences between and within groups. However, a more frequent blood sampling in the initial phase of the hyperinsulinemic clamp might have given us more accurate information on insulin suppression of lipolysis (Jensen and Nielsen, 2007).

During fasting, adipocyte ATGL content is upregulated (Nielsen et al., 2011) to mobilize metabolites for energy production as an alternative to glucose. We found an increased abundance of ATGL in abdominal adipose tissue in post- compared to the pre-menopausal women suggesting a higher capacity for lipolysis in post-menopausal women. Similarly, Jensen et al. found higher palmitate flux during estrogen-deficiency than after estrogen treatment of post-menopausal women (Jensen et al., 1994). Insulin stimulates adipose tissue triacylglycerol and fatty acid synthesis (Dimitriadis et al., 2011). As expected, we observed that insulin stimulation suppressed ATGL content in femoral adipose tissue in pre-menopausal women but, surprisingly, not in post-menopausal women. Theoretically, this could contribute to mobilization of FFA and ectopic fat deposition in post-menopausal women.

Recently, Dohlmann et al. found that mitochondrial DNA in adipose tissue is not changed after 6 weeks of high-intensity training but adipose tissue mitochondrial respiratory capacity was reduced (Dohlmann et al., 2018). In the present study, content of OXPHOS complex 5 in abdominal adipose tissue in post-menopausal women increased after 3 months of high-intensity training, but we observed no other increase in mitochondrial enzymes CS or OXPHOS complexes 1–5. Apparently, exercise training does not increase mitochondrial enzymes in human adipose tissue to the same extent as we have previously found in rat adipose tissue (Stallknecht et al., 1991). However, a new intriguing finding of the present study is that insulin decreased the content of OXPHOS complex 3 in both abdominal and femoral adipose tissue of both pre- and post-menopausal women after compared to before exercise training.

We sought to evaluate the effect of menopausal status on adipose tissue mass, glucose uptake, and protein content without obesity or aging as confounding factors. We thereby compromised on generalizability, as the included post-menopausal women were leaner than the general population. Our power calculations were based on a higher change in glucose uptake and adipose tissue reduction than we found in the study. Therefore, a type II error could explain our negative finding regarding glucose uptake. However, insulin-stimulated glucose uptake was comparable within groups in different subcutaneous depots as well as before and after the training intervention. A strength of the study is the investigation of *in vivo* insulin-stimulated glucose uptake in visceral and various subcutaneous adipose tissue depots via [18F]FDG PET/CT. Furthermore, extensive work to quantify adipose tissue protein content associated with glucose and lipid metabolism has been performed.

In conclusion, early menopause is not associated with increased insulin-stimulated glucose uptake in abdominal or femoral adipose tissue. However, post-menopausal women exhibited increased HK and ATGL content in abdominal adipose tissue suggesting increased capacity for adipose tissue glucose phosphorylation and lipolysis. Three months of high-intensity exercise training reduced subcutaneous and visceral adipose tissue masses similarly in pre- and post-menopausal women. This emphasizes that exercise training is important for mid-life women and should be encouraged.

## Data Availability Statement

The datasets generated for this study are available on request to the corresponding author.

## Ethics Statement

The studies involving human participants were reviewed and approved by the Ethical Committee in the capital region of Denmark, protocol nr. H-1-2012-150. The patients/participants provided their written informed consent to participate in this study.

## Author Contributions

BS, YH, AK, LE, JW, CM, JE, and MN substantially contributed to the conception or design of the work. CM, CR, CS, JE, and BU mainly contributed to the acquisition of data. ACl, ACh, and JK were the key-persons in the PET/CT data analysis (ACl and ACh) and the process of performing western blots (JK). All authors contributed significantly to the interpretation of data, and agreed to be accountable for all aspects of the work in ensuring that questions related to the accuracy or integrity of any part of the work are appropriately investigated and resolved, approved publication of the content, revised the manuscript critically, and added important intellectual content. JW and RF-S performed biochemical profiling. CR, JK, and JW performed western blotting. ACl, ACh, AK, and LE performed PET/CT. BU and ACh performed MRI. CS perfomred DXA-scans.

## Conflict of Interest

The authors declare that the research was conducted in the absence of any commercial or financial relationships that could be construed as a potential conflict of interest.

## Abbreviations

ACC: Acetyl-CoA carboxylase
AS160: Akt substrate 160 kDa (also known as TBC1D4)
ATGL: Adipose triglyceride lipase
BMI: Body mass index
CD36: Cluster of differentiation 36 [also known as FAT (fatty acid translocase)]
CS: Citrate synthase
DXA: Dual-energy X-ray absorptiometry
[^18^F]FDG: [^18^F]fluorodeoxyglucose
FFA: Free fatty acids
FSH: Follicle stimulating hormone
GLUT4: Glucose transporter 4
HK: Hexokinase
IDIF: Image derived input function
MRI: Magnetic resonance imaging
MR_glucose_: Metabolic rate of glucose
OXPHOS: Oxidative phosphorylation
PET/CT: Positron emission tomography/Computed tomography
SHBG: Sex hormone binding globulin
VOI: Volume of interest.
